# Randomised controlled trial of improvisational music therapy's effectiveness for children with autism spectrum disorders (TIME-A): study protocol

**DOI:** 10.1186/1471-2431-12-2

**Published:** 2012-01-05

**Authors:** Monika Geretsegger, Ulla Holck, Christian Gold

**Affiliations:** 1 Aalborg University, Faculty of Humanities, Department of Communication and Psychology, Aalborg Øst, Denmark; 2 University of Vienna, Faculty of Psychology, Department of Applied Psychology: Health, Development, Enhancement and Intervention, Vienna, Austria; 3 Grieg Academy Music Therapy Research Centre (GAMUT), Uni Health, Uni Research, Bergen, Norway

## Abstract

**Background:**

Previous research has suggested that music therapy may facilitate skills in areas typically affected by autism spectrum disorders such as social interaction and communication. However, generalisability of previous findings has been restricted, as studies were limited in either methodological accuracy or the clinical relevance of their approach. The aim of this study is to determine effects of improvisational music therapy on social communication skills of children with autism spectrum disorders. An additional aim of the study is to examine if variation in dose of treatment (i.e., number of music therapy sessions per week) affects outcome of therapy, and to determine cost-effectiveness.

**Methods/Design:**

Children aged between 4;0 and 6;11 years who are diagnosed with autism spectrum disorder will be randomly assigned to one of three conditions. Parents of all participants will receive three sessions of parent counselling (at 0, 2, and 5 months). In addition, children randomised to the two intervention groups will be offered individual, improvisational music therapy over a period of five months, either one session (low-intensity) or three sessions (high-intensity) per week. Generalised effects of music therapy will be measured using standardised scales completed by blinded assessors (Autism Diagnostic Observation Schedule, ADOS) and parents (Social Responsiveness Scale, SRS) before and 2, 5, and 12 months after randomisation. Cost effectiveness will be calculated as man years. A group sequential design with first interim look at N = 235 will ensure both power and efficiency.

**Discussion:**

Responding to the need for more rigorously designed trials examining the effectiveness of music therapy in autism spectrum disorders, this pragmatic trial sets out to generate findings that will be well generalisable to clinical practice. Addressing the issue of dose variation, this study's results will also provide information on the relevance of session frequency for therapy outcome.

**Trial Registration:**

Current Controlled Trials ISRCTN78923965.

## Background

Impairments or delayed development in skills concerning social interaction and communication are at the core of autism spectrum disorders (ASD) [1]. The attempts to help children with ASD develop meaningful language and social communication skills cover a wide range of different approaches. Yet, for most of the various intervention methods available and reported within ASD that are designed to improve communication and social interaction, only insufficient evidence of effectiveness exists [2]. Some favourable results can be found for approaches such as early behavioural interventions [3,4] and augmentative communication [5]. However, if methodologically rigorous standards are applied, statistically significant improvements in communication and speech could hitherto only be ascertained in a parent-mediated communication-focused intervention [6], parent-mediated behavioural interventions [2], and music therapy [2,7]. Similarly, in a review of "novel and emerging treatments" for ASD [8] including several nutritional supplements, diets, medications, and nonbiological treatments, it was found that the only treatment options that reached the highest ranking in an evidence-based grading system were melatonin, acetylcholinesterase inhibitors, naltrexone, and music therapy. Considering that pharmacological treatments typically target symptoms such as hyperactivity, agitation, or sleep disorders rather than core symptoms of ASD, and may have adverse effects [2,8], music therapy can be viewed as a promising, but not yet sufficiently evidenced treatment for improving social interaction and communication skills within ASD. Due to various methodological quality limitations of previous studies [2], further high quality randomised controlled trials (RCTs) on common interventions for ASD have been found to be urgently needed.

Music therapy has a long tradition within ASD [9,10], and there are many clinical reports, case studies, and single group studies (e.g. [11-13]; for an overview, see [14]) suggesting that music therapy may enhance skills of social communication such as initiating and responding to communicative acts. In recent years, increased efforts have been made to conduct more rigorous studies in this area. A Cochrane review combining the findings of three small controlled studies of music therapy in children with ASD [7] concluded that this type of intervention may have positive effects on the communicative skills of children with ASD, but also noted limited applicability of the studies' results to clinical practice due to very short duration of treatment conditions and low flexibility in music therapy techniques applied.

Following this review, some RCTs were conducted that strived for improved clinical relevance by applying treatment durations of several months as well as improvisational, flexible, child-centred methods of music therapy provided by trained therapists [15-17].

Improvisational music therapy for children with ASD may generally be described as a child-centred approach making use of the potential for social engagement and expression of emotions occurring through improvisational music making. Instead of practising targeted skills in an abstract manner, improvisational music therapy has been noted for its potential to provide a meaningful framework that, similar to early mother-infant interaction, encompasses relevant features of social communication such as being embedded in a shared history of interaction, having a common focus of attention, turn-taking, and musical and emotional attunement [15-18]. In the first of these newer RCTs on child-centred music therapy methods [15], it was suggested that improvisational music therapy may facilitate skills fundamental to social interaction in children with autism and proves to be effective in improving lower levels of initiating joint attention and responding to joint attention bids. Despite this trial's significant results, some methodological constraints such as its small sample size (N = 10) and large number of outcome measures limited the generalisability of its findings. Recent RCTs with slightly larger sample sizes of N = 23 (unpublished report, Thompson, McFerran, and Wigram, 2011) and N = 24 [16], respectively, also investigated effects of improvisational, child-centred music therapy approaches on social communication skills of young children with ASD, but were still seriously limited in sample size and test power. A large pragmatic RCT is needed to decide if improvisational music therapy improves core symptoms of ASD in a generalised setting.

### Objectives

The objectives of this study are as follows:

1.) To determine whether music therapy is superior to standard care in improving social communicative skills in children with ASD as assessed by independent clinicians at the end of the treatment period.

2.) To determine whether music therapy is superior to standard care in improving social responsiveness in children with ASD as assessed by parents/guardians at the end of the treatment period.

3.) To determine whether the response varies with variation of treatment intensity.

4.) To determine how the development of social communicative skills proceeds until follow-up twelve months after the start of treatment.

It is predicted that children's social communicative ability will increase over time, that social communicative skills may be better in music therapy conditions than in the standard care condition, and that more frequent music therapy may intensify the improvement in the skills assessed. Assessing participants' social communicative skills seven months after ending of treatment (12 months after randomisation) will yield important information on whether any effects in the skills investigated will be sustained.

## Methods/Design

### Participants

The study will include children referred from participating institutions (hospital departments, development centres, parents' support groups) who comply with the following criteria:

#### Inclusion criteria

*(a) Aged 4;0 to 6;11: *At their respective time of randomisation, the participants' age ranges between 4;0 and 6;11. Given the nature of basic social communication skills that are targeted in this study as occurring early in development, inclusion of young children is considered necessary; as it will be desirable for children to be able to attend therapy sessions without their parents, the lower age boundary was chosen based on the experience that children will usually be able to attend therapy in a one-to-one setting at that age; a further reason for setting the lower boundary at 4 years is that one of the scales measuring outcome (SRS, see below) is standardised for children from age 4. The upper age boundary was chosen in order to limit the sample to a group sharing similar everyday life conditions in preschool settings and/or around the time of transfer to school.

*(b) Diagnosis of autism spectrum disorder: *Participants must have a diagnosis of an autism spectrum disorder as assessed by a child psychiatrist or clinical psychologist according to ICD-10 criteria before their respective baseline assessment. Participants' diagnosis of ASD needs to be reconfirmed in the baseline assessment with children fulfilling diagnostic criteria for ASD on the Autism Diagnostic Observation Schedule (ADOS) [19] and on two of three domains of the Autism Diagnostic Interview-Revised (ADI-R) [20].

#### Exclusion criteria

*(a) Serious sensory disorder: *Children participating in the study must not be affected by serious sensory disorders such as blindness or deafness as this would alter the aim, course, and implementation of therapy.

*(b) Previous experience of music therapy: *Children having had music therapy sessions prior to study enrolment will not be included as this would be likely to have a strong influence on the course of therapy.

Non-verbal children as well as children with language skills may be included. Parents/guardians must give informed consent for their children to be involved in the study. Participants must be able to attend up to three weekly music therapy sessions. In cases where transportation to and from locations where the therapy sessions take place might be difficult to provide by parents/guardians, travelling allowances may be made available to avoid bias or drop-out due to any family's financial restrictions.

### Baseline assessment

To support the diagnosis of autism, and to establish a baseline of the respective outcome measure, the Autism Diagnostic Observation Schedule (ADOS) [19] will be administered to potential participants. Additionally, the Autism Diagnostic Interview-Revised (ADI-R) [20] will be administered to parents/guardians to acquire data not only on the behaviour displayed during baseline assessment, but also on the history of development of each child, and to avoid loss of specificity [21,22]. The children's level of cognitive ability will be assessed using the Kaufman Assessment Battery for Children (K-ABC) [23]. In cases where the application of the K-ABC is not possible due to the respective child's limitations in complying with the requirements of the testing situation, children's level of cognitive functioning will be estimated by the assessor to fall into one of three categories (no mental retardation vs. mild mental retardation vs. moderate to profound mental retardation according to ICD-10 criteria) using clinical judgment. To establish the baseline of the secondary outcome measure, parents/guardians will be asked to complete the Social Responsiveness Scale (SRS) [24]. In addition, standard demographic parameters (gender, age, first language, family size, parents' educational background), comorbidities, and information on concomitant treatment will be recorded.

### Interventions

Participants will be randomly assigned to one of the following three conditions:

*(1) High-intensity music therapy: *Improvisational music therapy sessions in an individual setting thrice weekly for five months (i.e., a total of up to 60 sessions, depending on possible omission of single sessions due to sickness or holidays), and three sessions of parent counselling as a "standard care condition" (one session at baseline, one after two months, and a third one after five months).

*(2) Low-intensity music therapy: *Improvisational music therapy sessions in an individual setting once a week for five months (i.e., a total of up to 20 sessions), and three sessions of parent counselling (at baseline, two months, and five months).

*(3) Standard care: *Three sessions of parent counselling (at baseline, after two months, and after five months).

#### Concomitant treatment

Any concomitant treatment or therapeutic interventions that participating children might receive outside the trial will be recorded during assessment sessions before randomisation and after 2, 5, and 12 months, specifying the kind and amount or frequency of intervention.

We consider a treatment duration of five months to be sufficient for detectable developments in children's social communication skills. Some of the earlier RCTs on music therapy in autism [15-17] were able to identify effects with shorter or similar duration; see also a meta-analysis of the dose-effect relationship in music therapy [25]. Additionally, we believe this time frame not to be overly long for being able to sustain parents'/guardians' motivation to participate in the study.

#### Description of music therapy

The duration of music therapy sessions will be 30 minutes. Therapists conducting the music therapy sessions will be trained music therapists (master's level or equivalent) with clinical experience of working with children with ASD.

The music therapy approach applied in this study is based on the ideas and principles of improvisational music therapy [26,27], findings from previous music therapy research [13,15-17,28], and developmental psychology [29]. The music played or sung by the therapist is generally attuned to the child's (musical or other) behaviour and expression and includes various improvisational techniques to engage the child and establish contact with the child. To this end, "musical" features of the child's expression (pulse, rhythmic, dynamic or melodic patterns, timbre etc.) may be mirrored, reinforced, or complemented, thus allowing for moments of synchronisation between child and therapist and giving the child's expressions a pragmatic meaning within the context. To allow elicitation of specific social communicative behaviours, the therapist may also gently provoke the child e.g. by violating expectations or jointly developed patterns. While engaging in joint musical activities within a shared history of interaction, the child is offered opportunities to develop and enhance skills such as affect sharing, joint attention, imitation, reciprocity, and turn-taking, all of which are associated with later development in language and social competency [30,31].

#### Description of parent counselling

Parent counselling sessions will be approximately 60 minutes and will be conducted by a music therapist and/or clinical psychologist experienced in the field of ASD. Counselling sessions will comprise supporting conversations with a focus on current concerns, problems, and difficulties arising from the child's diagnosis, behaviour, and development over time as well as providing information about ASD, child development, and social communication relevant to the families' everyday life situations.

#### Treatment guide for music therapy and parent counselling

Music therapy and parent counselling sessions will both be provided in accordance with a treatment guide devised for this study in order to specify the treatment procedures and to allow for training of staff and replication of treatment. Within this guide, the setting, general goals, and basic principles of the intervention as well as exemplifications will be outlined. The guidelines are to be administered flexibly according to the requirements of the respective situation and needs of the client or parent within the therapy process or counselling session and can only be applied in combination with and relying on the clinical expertise of the therapist or counsellor.

While the treatment guide will help to ensure the trial's validity and replicability, it is also important to retain flexibility and openness to emerging procedures within music therapy in clinical practice [32]. Keeping enough "space" for flexible adaptation within the treatment guide according to the child's spontaneous social behaviours will also ensure that the intervention will be shaped in a way that is tailored to the individual strengths and needs of each child, thus addressing the great variability of developmental profiles present in children on the autism spectrum. The treatment guide will be described in a separate paper.

#### Assessment of treatment fidelity

To determine if the treatment is conducted as intended, fidelity check measures will be applied as follows: after every session, the therapist/counsellor will document significant events, notable child/parent behaviours, and interventions applied. In addition to these self-reports, all therapy and parent counselling sessions will be videotaped to allow for assessment by independent raters [33]. As in a previous RCT in music therapy [34], adherence to the method and competence in its application will also be monitored and sustained through clinical supervision of music therapists/counsellors, utilising the therapists' clinical notes and video-recordings of sessions where necessary.

### Study design

**Figure 1:**
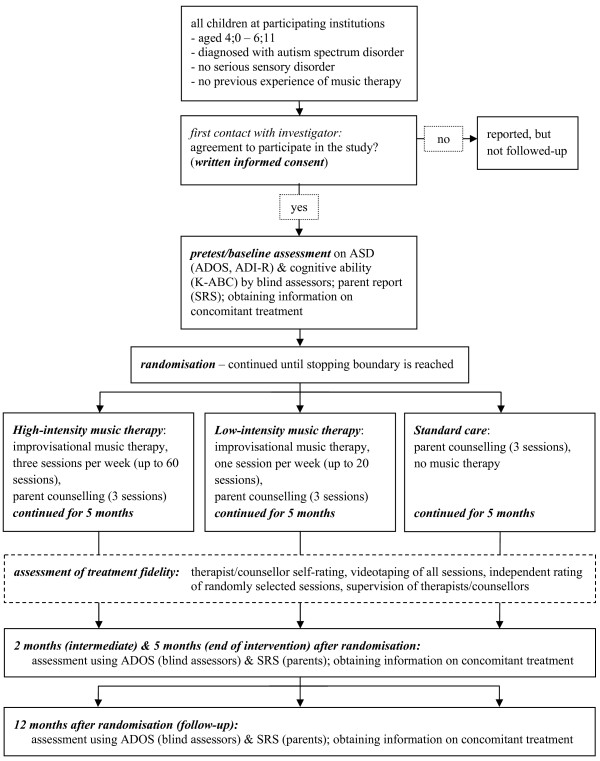
**Flow chart of the study design**. *Abbreviations: *ASD - autism spectrum disorders; ADOS - Autism Diagnostic Observation Schedule; ADI-R - Autism Diagnostic Interview-Revised; K-ABC - Kaufman Assessment Battery for Children; SRS - Social Responsiveness Scale.

The study will be a pragmatic international multicentre single-blind (assessor-blinded) randomised controlled trial with three parallel arms. After inclusion in the study and baseline assessment, participants will be assigned to one of the music therapy conditions or the standard care condition on an individual basis according to a computer generated randomisation list. The allocation ratio of intended numbers of participants in the comparison groups will be 1:1:2 so that the number of children receiving music therapy will be similar to the number of participants in the standard care condition. To this end, randomisation will be made in blocks with random sequences of block sizes of 4 or 8 respectively (a separate list for each site) to avoid possible guessing of some allocations. Before random assignment is performed, it has to be confirmed by the investigator recruiting participants that the eligibility criteria have been met and participants are formally enrolled. Once recruitment and data collection at baseline are complete and informed consent to participate in the study by the parents/guardians has been obtained, the respective randomisation code will be revealed to the investigator by an administrative person at the central randomisation office who will have no contact to participants. An overview of the study design is shown in Figure 1.

### Power calculation and sample size

#### Estimate of effect size

A Cochrane review on music therapy for ASD found effect sizes (standardised mean differences) of d = 0.50 and 0.36 for gestural and verbal communicative skills, respectively [7]. However, these figures were derived from small studies with low precision and using unpublished scales [7]. A more precise estimate using the ADOS scale may be derived from the PACT trial [6] where an effect of d = 0.24 was found. That study was more similar in design to the present study but used a different behavioural intervention. In summary, an effect size in the small (d = 0.20) to medium (d = 0.50) range may be expected [35], corresponding to a 1 to 2.5 points difference on the ADOS scale that typically has SD = 5 [6]. An effect size in this range would be clinically meaningful as the ADOS scale measures the core symptoms of ASD which are difficult to influence with any treatment.

#### Parameters for sample size calculation

The two music therapy arms (high- and low-intensity) will be taken together for the primary analysis to answer the question if music therapy in general is superior to standard care. Half of all participants will be assigned to music therapy. In light of the uncertainty around the true effect size and the difficulty of recruiting large samples, a group sequential design (GSD, [36]) will ultimately ensure 80% power (two-sided alpha 0.05) even for a small effect, while avoiding excessively large sample size if there is in fact a medium effect. We used the common Lan-DeMets alpha spending function and a Pocock boundary to make early stopping likely. Calculations and simulations were made for up to four equally spaced looks, using East 5.4 software by Cytel Inc., 2010.

#### Results

Table 1 shows that if there is a medium effect of music therapy, power at the first interim look (usable N = 235) will be 93%. Power will also be acceptable (76%) if the effect is slightly smaller than medium. If there is only a small effect size, power can still be retained by recruiting more participants. An independent data monitoring committee will perform the interim look. We will aim to randomise N = 300 participants (150 to standard care and 75 to each type of music therapy) to account for possible drop-outs and clustering within sites [37]. However, the actual interim look may be taken at a different sample size, depending on recruitment progress and funding.

**Table 1:** Sample size and power under different scenarios

Raw difference on the ADOS scale (SD = 5)	Standardised effect size (Cohen's d)	Power at first interim look^1^	Test power over all 4 interim looks^2^
2.5	0.5 (medium effect)	93%	100%

2	0.4	76%	100%

1.5	0.3	47%	99%

1	0.2 (small effect)	20%	80%

Note. Based on 10000 simulations for each scenario. ^1^Usable N = 235 (300 randomised). ^2^Maximum usable N = 939.

### Outcomes

The study will use assessments by blinded clinicians as well as reports by parents/guardians. Outcome variables will be assessed at several time points: after the baseline assessment (taking place before each individual's randomisation), the outcome measures will be reapplied after an interval of two months (intermediate), once again after an interval of five months (end of intervention), and finally at follow-up twelve months after randomisation. Thus, requirements such as close analysis of the mechanism of action (i.e. how long an intervention is required to begin to have an effect) and investigation on maintenance of any observed changes over a longer period of time [2] can be met.

#### Primary outcome

To allow for potential comparison with studies investigating similar interventions as well as for potential inclusion in later reviews, a validated scale widely used in research and academic practice will be used for assessing social communication skills: The Autism Diagnostic Observation Schedule (ADOS) is a semi-structured, standardised observation instrument designed to assess communication, social interaction, and play or imaginative use of materials using 28 to 31 specific behavioural criteria in one of four modules chosen individually depending on the respective child's expressive language level and chronological age [19]. Inter-rater reliability, test-retest reliability, and internal validity have been demonstrated for the ADOS [19]. The ADOS is viewed especially suitable for this study considering that, although standardised, its assessment is based on play-based interactions between assessor and child, thus similar in its setting to the music therapy situation. This study's primary outcome will be the ADOS social communication algorithm score which has been used as an outcome measure in previous RCTs investigating effects of interventions for autism [6,38,39]. In order to improve sensitivity to change, the scoring procedure will be modified as in an earlier RCT on treatment effects [6], i.e. the module applied to each child will be the same across assessment points (instead of adjusting the module to potentially developed expressive language skills) to avoid discontinuity of scores, and the full range of scores will be retained (instead of recoding 3's to 2's as in the standard diagnostic algorithm) [40].

To avoid bias in observation and assessment, assessors administering the instrument will be blinded to group allocation of the children to be assessed. This will be secured for example by having the assessments take place in another location than music therapy sessions. Success of blinding will be verified by asking assessors whether or not they inadvertently found out about the child's allocation.

#### Secondary outcomes

In order to supplement the assessment, parents/guardians will be asked to complete the Social Responsiveness Scale (SRS) [24] at baseline, and two, five, and twelve months after randomisation. The 65-item rating scale measures the severity of autism spectrum symptoms occurring in natural social settings, assessing social awareness, social information processing, capacity for reciprocal social communication, social anxiety/avoidance, and autistic preoccupations and traits. Defined as suitable for assessing treatment response [41], these five subscales seem appropriate as secondary outcome measures. The scale features high inter-rater and test-retest reliability as well as internal validity rates and may be completed in 15 to 20 minutes [42-44], thus easily applicable during appointments.

Cost-effectiveness of music therapy will be compared to standard care. Cost will be measured as real resources used in treatment, in terms of personnel hours of work. Effectiveness is measured by ADOS, and cost-effectiveness ratios and incremental cost-effectiveness ratios for the different alternatives can be calculated. Gains for the general health care sector and society will be more long term, and can hardly be included in this project. However, some consideration will be made as to possible effects on school attainment. Costs can be made comparable across countries using purchasing power parity measures.

### Statistical analyses

The primary analysis will be undertaken on an intention-to-treat basis, and two-sided tests will be applied at a 5% alpha level. Following assessment of normality, treatment effects will be analysed using a generalized estimating equations (GEE) approach that allows for analysis of longitudinal data while accounting for the correlation among the repeated observations for each subject [45]. GEE analyses will also be used to examine dose-effect relationships and to explore possible confounding effects of site or relevant subgroups such as age or ASD subtype.

### Ethical issues

The study protocol was approved by the Faculty of Humanities' Human Research Ethics Board (HREB) at Aalborg University, Denmark. Freely given, written informed consent will be obtained from participant's parents/guardians prior to study enrolment in accordance with regulatory requirements. Random allocation of participants to study groups is considered reasonable as no adverse effects are expected in any of the conditions. Inconveniences caused by the necessity to attend three weekly sessions of music therapy for the families assigned to this study group are considered tolerable in view of the anticipated benefit for the child receiving therapy.

## Discussion

High clinical applicability of this RCT's findings is to be achieved through therapy conditions that are close to clinical practice in terms of broad eligibility criteria (verbal and non-verbal children, all types of ASD), treatment duration (several months), and therapy techniques (improvisational music therapy conducted by experienced therapists in a typical setting).

The study's limitations are also its strengths: The absence of outcomes proximal to music therapy [15,17]; the heterogeneity of standard care as a control condition; and the heterogeneity of the population [46] may be seen as limitations, but are features that will improve feasibility and that are in line with pragmatic trials of effectiveness whose focus is to help users choose between options [47].

Conclusions that will emerge from this study are expected to contribute to the evidence base of treatment options for children with ASD. The results of the trial will provide evidence on the effectiveness of music therapy as a treatment for ASD and will also provide information on the relevance of session frequency for therapy outcome. Furthermore, findings gained through the application of a treatment guide within this study may help to further specify music therapy treatment guidelines for this population and to enrich future training and education of music therapists and other health care professionals working in the field of ASD.

Findings of this study will also be relevant for other fields where music therapy is applied, such as adult mental health [10,25], and for basic research into the musical qualities of early mother-infant communication providing a rationale for music therapy [18,29]. Music therapy in general is an intervention that focuses on developing social and emotional abilities, and ASD is a case in point because impairments in these abilities are central for ASD.

## Competing interests

MG, UH, and CG are clinically trained music therapists.

## Authors' contributions

MG and CG conceived the study and developed the study design. MG drafted the manuscript. CG did the power calculation and helped to draft the manuscript. UH contributed to the development of the study design and helped to draft the manuscript. All authors read and approved the final manuscript.

## Pre-publication history

The pre-publication history for this paper can be accessed here:

http://www.biomedcentral.com/1471-2431/12/2/prepub
